# A strategically designed small molecule attacks alpha-ketoglutarate dehydrogenase in tumor cells through a redox process

**DOI:** 10.1186/2049-3002-2-4

**Published:** 2014-03-10

**Authors:** Shawn D Stuart, Alexandra Schauble, Sunita Gupta, Adam D Kennedy, Brian R Keppler, Paul M Bingham, Zuzana Zachar

**Affiliations:** 1 Graduate Program in Genetics, Department of Molecular Genetics and Microbiology, Stony Brook University, Stony Brook, NY 11794, USA; 2 Department of Biochemistry and Cell Biology, Stony Brook University, Stony Brook, NY 11794, USA; 3 Cornerstone Pharmaceuticals, Inc, Stony Brook, NY 11794, USA; 4 Cornerstone Pharmaceuticals, Inc, Cranbury, NJ 08512, USA; 5 Metabolon, Inc, Research Triangle Park, Durham, NC 27709, USA

**Keywords:** Alpha-ketoglutarate dehydrogenase, Cancer metabolism, Chemotherapy, Glutathionylation, Lipoate, Pyruvate dehydrogenase, ROS

## Abstract

**Background:**

Targeting cancer cell metabolism is recognized as a promising arena for development of cancer chemotherapeutics. Moreover, redox metabolism is also systematically altered in tumor cells. Indeed, there is growing reason to believe that tumor-specific alteration of redox control of metabolism will be central to understanding and attacking malignancy. We report here that lipoate analog CPI-613 attacks a gate-keeping, lipoate-using metabolic enzyme, alpha-ketoglutarate dehydrogenase (KGDH), by a redox mechanism selectively in tumors cells.

**Results:**

CPI-613 inhibited KGDH function strongly and rapidly, selectively in tumor cells. Moreover, CPI-613 induced a correspondingly rapid, powerful redox signal in tumor cell mitochondria. This signal was associated with redox modification of KGDH (including extensive enzyme glutathionylation and redox blockage of enzyme lipoate sulfhydryls), correlating with KGDH inactivation. The source of this tumor-specific mitochondrial redox modulatory signal was not electron transport complexes (I or III), but was largely or entirely the E3 (dihydrolipoamide dehydrogenase) component of dehydrogenases, including KGDH. Finally, we demonstrated that KGDH activity was redox regulated (in tumor cells), as expected if a tumor-specific redox process (auto)regulates KGDH.

**Conclusions:**

Our data demonstrate that lipoate analog CPI-613 attacks redox control of KGDH activity in tumor cells, perhaps by modulation of an existing lipoate-sensitive allosteric process normally governing tumor cell KGDH activity. Together with its previously reported, mechanistically distinct (non-redox) effects on the other major, lipoate-using mitochondrial metabolic enzyme, pyruvate dehydrogenase, CPI-613’s KGDH effects indicate that this agent simultaneously attacks multiple central, essential components of tumor cell metabolic regulation.

## Background

Reactive oxygen species (ROS) have emerged as potent signaling molecules with the ability to modulate a number of cellular signaling processes, owing to their ability to modify proteins, including via oxidation of specific cysteine residues [1,2]. ROS have also been found to directly regulate key enzymes of matter/energy metabolism. The mitochondrial tricarboxylic acid (TCA) cycle enzyme aconitase has long been known to be inactivated by ROS through its iron cluster [3]. Moreover, there is evidence for redox-dependent changes associated with malignancy-related metabolic alterations in breast cancer development [4]. In addition, the glycolytic enzymes glyceraldehyde 3-phosphate dehydrogenase [5] and the tumor-specific M2 splice variant of pyruvate kinase [6-8] have both been shown to be inhibited by the oxidation of specific cysteine residues, apparently redirecting carbon flux through the pentose phosphate pathway and away from the glycolytic pathway to generate ROS-detoxifying reducing potential.

**Figure 1:**
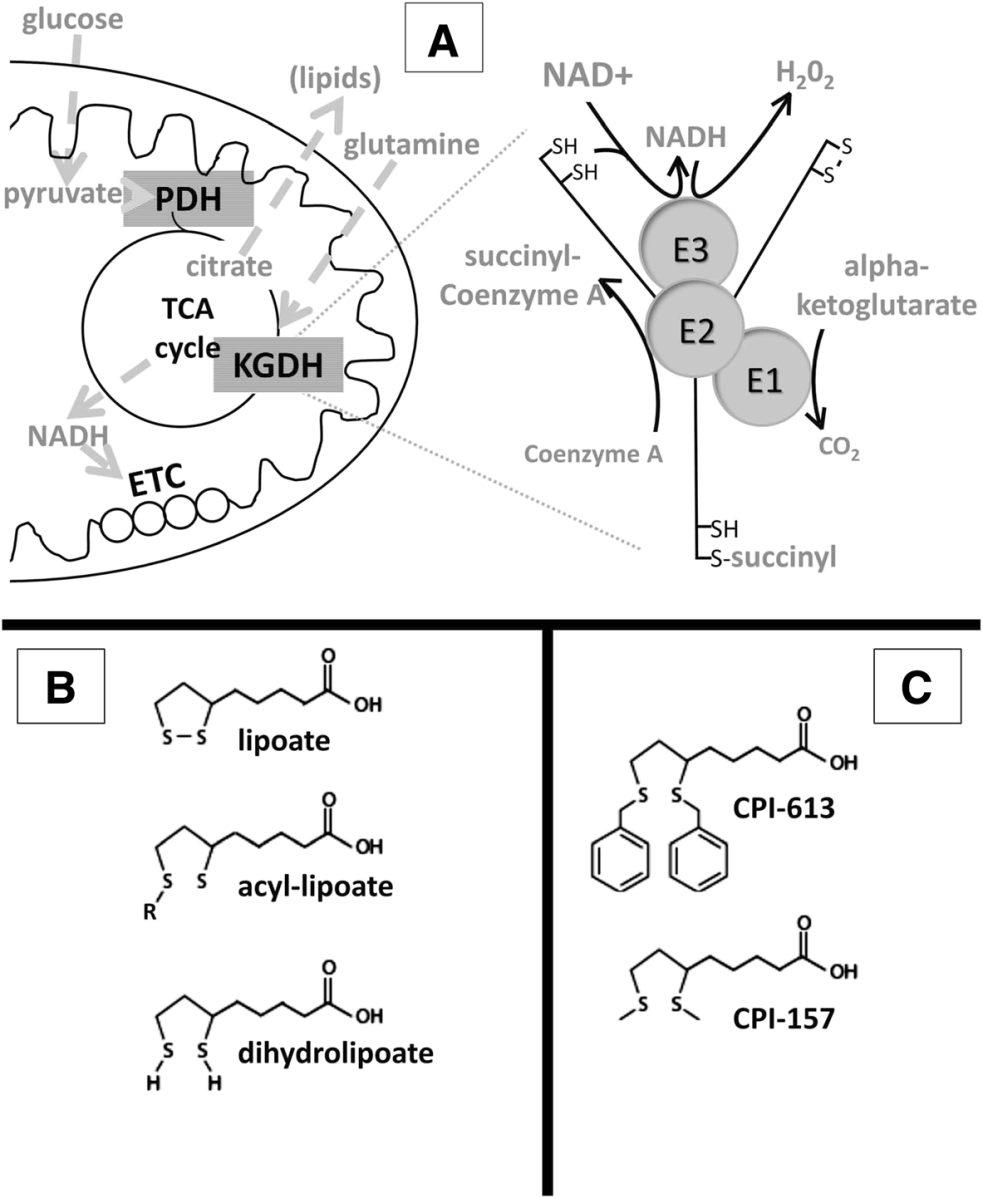
**Role of α-ketoglutarate dehydrogenase and its lipoate residues. (A)** On the left are selected details of mitochondrial metabolism, including the matrix TCA cycle and anabolic diversion of TCA citrate in support of cytosolic lipid biosynthesis. KGDH is an enzyme complex in the TCA cycle. Entry of glutamine-derived carbon into the TCA cycle is controlled by KGDH. Features of the structure and catalytic reactions of the KGDH complex are illustrated on the right. There are two sources of reducing potential for the catalysis of H_2_O_2_ production by E3: reduced lipoate (forward reaction) and NADH (reverse reaction). **(B)** Chemical details of the three intermediates in the natural lipoate catalytic cycles of KGDH and pyruvate dehydrogenase are shown. R indicates a succinyl residue in KGDH and an acetyl residue in pyruvate dehydrogenase. **(C)** Selected details of the structures of the two lipoate analogs used in these studies are shown. KGDH, α-ketoglutarate dehydrogenase; TCA, tricarboxylic acid.

The heightened need in cancer cells for biosynthetic intermediates [9-12] results in increased use of a ‘truncated’ TCA cycle, including diversion of citrate to cytosolic export for use in lipid synthesis (Figure 1A). To replenish TCA cycle intermediates in support of such anabolic processes, cancer cells rely disproportionately on glutamine, which enters the cycle as α-ketoglutarate via the α-ketoglutarate dehydrogenase (KGDH) complex ( [13] and Figure 1A).

TCA cycle enzymes are tightly regulated and their activities and regulation are often substantially altered in cancer cells. For example, the pyruvate dehydrogenase (PDH) regulatory kinases (PDKs 1 to 4), negative regulators of the PDH complex, are up-regulated in many cancers, apparently to control anabolic flux and to modulate mitochondrial O_2_ consumption in the hypoxic tumor environment [14-17]. Further, results presented herein indicate tumor-specific alteration of KGDH regulation. These generalized alterations of metabolic regulation of cancer cell mitochondrial metabolism represent potential targets for next-generation chemotherapeutics.

The KGDH and PDH complexes sit at the center of mitochondrial metabolism, generally controlling the large majority of carbon flow into the TCA cycle, derived from glutamine and pyruvate, respectively. PDH and KGDH are among the small group of enzymes using lipoate as a catalytic cofactor, a role in which lipoate is also known to generate information addressing tumor-specific alterations in PDH regulation (references above; reviewed in [18,19]). Thus, we have explored the use of lipoate analogs as cancer chemotherapeutic agents.

In contrast to PDH, KGDH is not regulated by phosphorylation and has previously been assumed to be controlled primarily by allosteric processes [20]. Evidence is emerging, however, of additional, redox-mediated mechanisms of KGDH regulation. Applegate and colleagues [21] report reversible inactivation of KGDH in isolated mitochondria treated with hydrogen peroxide, with this inactivation resulting from glutathionylation of the E2 lipoate residues. Intriguingly, the E3 (dihydrolipoamide dehydrogenase) subunit of KGDH is also now recognized as a major source of mitochondrial ROS [22-24], although many details of this process remain to be defined.

We previously described a member of a novel class of anti-cancer lipoate derivatives, (CPI-613, Figure 1C) that specifically induces inactivating phosphorylation of tumor cell PDH via stimulation of PDKs 1 to 4. This CPI-613-induced PDH inactivation contributes to the collapse of mitochondrial function and the activation of multiple tumor cell death pathways [18]. In addition, we reported very strong CPI-613 tumor growth inhibition in two human xenograft mouse models, indicating *in vivo* efficacy (ibid.). CPI-613 is in early clinical trials, showing a strong safety profile and some early, anecdotal indications of efficacy [25].

We report here the novel effects of CPI-613 on the second lipoate-containing, mitochondrial enzyme complex, KGDH. CPI-613 induces a large, tumor-specific burst of mitochondrial ROS, apparently from the E3 subunit of the KGDH complex itself. CPI-613 appears to hyper-stimulate an endogenous, redox mechanism for KGDH autoregulation in a tumor-specific fashion. This ROS signal inhibits KGDH activity with associated glutathionylation of enzyme sulfhydryls, and redox modification of the endogenous lipoate residues of the KGDH E2 subunit. Combined with its mechanistically distinct effects on PDH, this CPI-613-induced inhibition of KGDH contributes to powerful tumor-specific inhibition of mitochondrial metabolism. Thus, this single drug simultaneously and independently attacks two central, essential metabolic multi-enzyme complexes, including KGDH, which may occupy a previously unexplored interface between tumor-specific redox regulation and matter/energy metabolism.

## Methods

### Cell culture

The human non-small cell lung carcinoma cell line NCI-H460 and pancreatic carcinoma cell line BxPC-3 were purchased from the American Type Culture Collection (Manassas, VA, USA) and cultured in Roswell Park Memorial Institute (RPMI)-1640 medium supplemented with 10% fetal bovine serum, 100 units/ml penicillin and 100 μg/ml streptomycin (Life Technologies, Carlsbad, CA, USA) unless otherwise indicated.

Normal human bronchial/tracheal epithelial (HBT) cells were purchased from Lifeline Cell Technology (Walkersville, MD, USA) and were propagated according to the supplier’s instructions in media developed by and obtained from the supplier. Experiments reported used normal cells at passages six to ten.

H460 cells lacking mitochondrial DNA (ρ°) were derived as described previously [26].

### Chemicals

Highly purified CPI-613 and CPI-157 were synthesized from D,L lipoate as described previously [18]. N-acetylcysteine (NAC), auranofin, resazurin, diaphorase, glutaredoxin-1, reduced glutathione, Triton X-100, digitonin, lauryl maltoside, dithiothreitol (DTT), NAD^+^, ADP, thiamine pyrophosphate, coenzyme-A (CoA), and N-ethylmaleimide (NEM) were purchased from Sigma-Aldrich (St. Louis, MO, USA). Biotin-HDPD and gel filtration columns (PD10) were from Thermo Scientific (Waltham, MA, USA). 2',7'-dichlorodihydrofluorescein diacetate (DCF), dihydroethidium (DHE), and Amplex Red were from Life Technologies. Antibodies to Prx1, Prx3 and reduced lipoate were purchased from AbCam (Cambridge, MA, USA). Antibodies against dihydrolipoamide dehydrogenase (E3) were from Rockland Immunochemicals (Gilbertsville, PA, USA) and KGDH dihydrolipoamide succinyltransferase (E2) antibodies were from Cell Signaling (Danvers, MA, USA).

### ATP assay

Total cellular ATP levels were measured using CellTiter-Glo luminescence assay (Promega, Madison, WI, USA) according to manufacturer’s directions.

### Assessment of mitochondrial ATP production from different carbon sources

H460 cells were seeded at 10,000 cells per well in black, clear bottom, 96-well plates in RPMI (11 mM glucose, 2 mM glutamine) medium and grown overnight. The medium was then changed to RPMI without glucose and containing 10 mM pyruvate and 2 mM glutamine alone or together with 0.1 mM water soluble oleic acid (Sigma-Aldrich). After 24 hours, the medium was replaced with fresh RPMI without glucose and containing either 10 mM pyruvate and 2 mM glutamine or 0.1 mM oleic acid and 0.5 mM aspartate (matched to overnight adaptation) and containing CPI-613 (240 μM) in the treated samples or solvent alone in the controls for 2 hours before ATP level measurements.

Cells in these final media without drug treatment retain stable and robust ATP production (exclusively mitochondrial) for more than 6 hours, with pyruvate plus glutamine and oleic acid generating comparable ATP outputs.

### Small interfering RNAs

Small interfering RNA (siRNA) duplexes against dihydrolipoamide dehydrogenase (E3) were purchased from IDT (Coralville, IA, USA) with the following sequences: 5’-CCUGUGAAGAUAUAGCUA, 5’-CAGACUCUAGCUAUAUCU. siRNA duplexes were transfected into NCI-H460 cells using Lipofectamine 2000 (Life Technologies) as per manufacturer’s instructions.

### CO_2_ release through carbon source oxidation

Oxidative release of carbons as carbon dioxide from glutamate was assayed by filter capture as described in [4] with minor modifications. We seeded 48-well plates with 100,000 cells per well in 0.5 ml of medium. After 18 to 25 hours, the medium was replaced with fresh medium containing drug solvent (dimethyl sulfoxide) alone or with CPI-613 for time intervals and drug concentrations as indicated. For the last 30 minutes of incubation, 0.3 μCi of radiolabeled substrate was added. At termination, 75 μl of 3 M perchloric acid was added to each well and wells were immediately covered with phenylethylamine-saturated 3 mm discs to capture released CO_2_. After 24 hours, discs were transferred into scintillation vials containing 1 ml of Biosafe-II scintillation cocktail (Research Products, International, Mount Prospect, IL) and counted.

### Quantitation of intracellular reactive oxygen species levels

H460 cells were plated in 35 mm tissue culture dishes at a density of approximately 300,000 cells and grown overnight. After 16 to 20 hours, drug or vehicle control was added for specified times. For the final 15 minutes of drug treatment, 5 μM DCF or dihydroethidium (DHE) was added. Cells were then detached via trypsinization and collected for fluorescence-activated cell sorting (FACS) analysis on a FACScalibur flow cytometer (BD, Franklin Lakes, NJ, USA) using CellQuest Pro software.

### Steady state metabolite levels determination

In brief, samples were extracted and split into equal parts for analysis on the gas chromatography-mass spectrometry and liquid chromatography-tandem mass spectrometry platforms [27]. Proprietary software was used to match ions to an in-house library of standards for metabolite identification and for metabolite quantitation by peak area integration [28].

Extracts were prepared according to Metabolon’s (Durham, NC, USA) standard methanol-based extraction protocol [27]. Samples were analyzed on a Thermo-Finnigan Trace DSQ fast-scanning single-quadrupole mass spectrometer (Waltham, MA) using electron impact ionization. (For additional technical details on cell preparation, techniques and statistical analysis, see Additional file 1).

### Western blot analysis

For western blot analysis, 2X lithium dodecyl sulfate (LDS) loading buffer (500 mM Tris (pH 8.5), 4% LDS, 20% glycerol, 1 mM EDTA, 0.44 mM SERVA Blue G250, 0.35 mM Phenol Red, plus 100 mM DTT unless otherwise noted) was added to samples followed by heating at 70°C for 10 minutes. Proteins were separated via SDS-PAGE then transferred to polyvinylidene difluoride membranes and detected via chemiluminescence using the WesternBreeze detection kit (Life Technologies).

### Detection of glutathionylated proteins

Glutathionylated proteins were detected as described previously with modifications [21]. Briefly, cells were treated for 3 hours with CPI-613, washed with PBS then treated with ice-cold N-buffer (40 mM HEPES (pH 7.4), 50 mM NaCl, 1 mM EGTA, 1 mM EDTA, plus Pierce protease inhibitor cocktail) containing 100 mM NEM for 5 minutes to alkylate free sulfhydryls. Mitochondria were then purified as described previously [29] and permeabilized with 0.05% Triton X-100. Unreacted NEM was scavenged with 1 mM NAC followed by treatment with 2.0 units/ml glutaredoxin and 1.4 mM reduced glutathione to deglutathionylate protein cysteines. Free cysteines were then labeled with 1.6 mM biotin-HPDP for 5 minutes followed by addition of 2 mM NAC to scavenge unreacted biotin-HPDP. Biotin-HPDP-NAC was removed by gel filtration and streptavidin-conjugated Dynabeads (Life Technologies) were used to capture biotinylated (glutathionylated) proteins as per manufacturer’s instructions. Beads were then mixed with 2X LDS loading buffer containing 100 mM DTT to release captured proteins followed by western blot analysis.

### Lipoate protection assay

Cells were washed twice with ice-cold PBS before exposure to ice-cold N-buffer containing 10 mM NEM to block non-derivatized lipoates. Following a 5 minute incubation on ice, CHAPS was added at a final concentration of 1% to lyse cells. Lysates were transferred to 1.5 ml microfuge tubes and incubated on ice for an additional 5 minutes with occasional vortexing followed by centrifugation at 15,000x g for 10 minutes to pellet insoluble material. Supernatants were then mixed 1:1 with 2X LDS loading buffer containing DTT at a final concentration of 100 mM to reverse oxidative modifications of lipoate (including removal of glutathione residues) and analyzed via SDS-PAGE and western blot with antibody against non-derivatized lipoate.

### Hydrogen peroxide production assay

Hydrogen peroxide production by purified porcine KGDH (Sigma-Aldrich) *in vitro* was measured using the Amplex Red hydrogen peroxide assay kit (Life Technologies) according to manufacturer’s instructions and as described in [22].

### Peroxiredoxin assay

Peroxiredoxin oxidation status was assayed as described previously [30] with minor modifications. Following treatment, cells were washed twice with ice-cold PBS then incubated with ice-cold N-buffer containing 100 mM NEM for 10 minutes on ice. CHAPS was added at a final concentration of 1% and incubated on ice for a further 10 minutes with shaking. Samples were centrifuged for 10 minutes at 15,000x g to pellet insoluble material. Supernatant was combined with 2X loading buffer (without DTT) and proteins were resolved via SDS-PAGE under oxidizing conditions and probed via western blot.

### *In vitro* analysis of α-ketoglutarate dehydrogenase activity

Cells grown on solid substrate in the same 48-well plate format as employed for flux analysis (either treated or untreated with CPI-613) were lysed for 2 minutes at room temperature with 0.03% digitonin in PBS to selectively disrupt plasma membranes, releasing cytosolic nicotinamide coenzymes and carbon sources. This initial lysis solution was replaced with mitochondrial lysis butter (0.5% lauryl maltoside, 50 mM Tris (pH 7.4) and 1 mM MgCl_2_) for two minutes. Twelve-minute reactions were initiated by adding a 10X buffer to the mitochondrial lysates, producing the following final component concentrations: 0.6 mM or 0 mM α-ketoglutarate; 50 μM CoA; 225 μM thiamine pyrophosphate; 250 μM NAD^+^; 50 μM ADP; 15 μM glutathione; 15 μM resazurin; 0.5 units/ml diaphorase. NAD^+^ reduction was assayed by resazurin reduction as above. Reaction rates were linear over this reaction time.

To further investigate the role of redox modification of KGDH in drug-induced inhibition of KGDH, 10 mM DTT was added to both lysis buffers in duplicate reactions.

### Statistical analysis

Except where otherwise noted, Student’s t-test was used for data analysis. *P* <0.05 was considered significant. All error bars are standard error of the mean (SEM).

## Results

### CPI-613 induces a strong mitochondrial burst of reactive oxygen species

**Figure 2:**
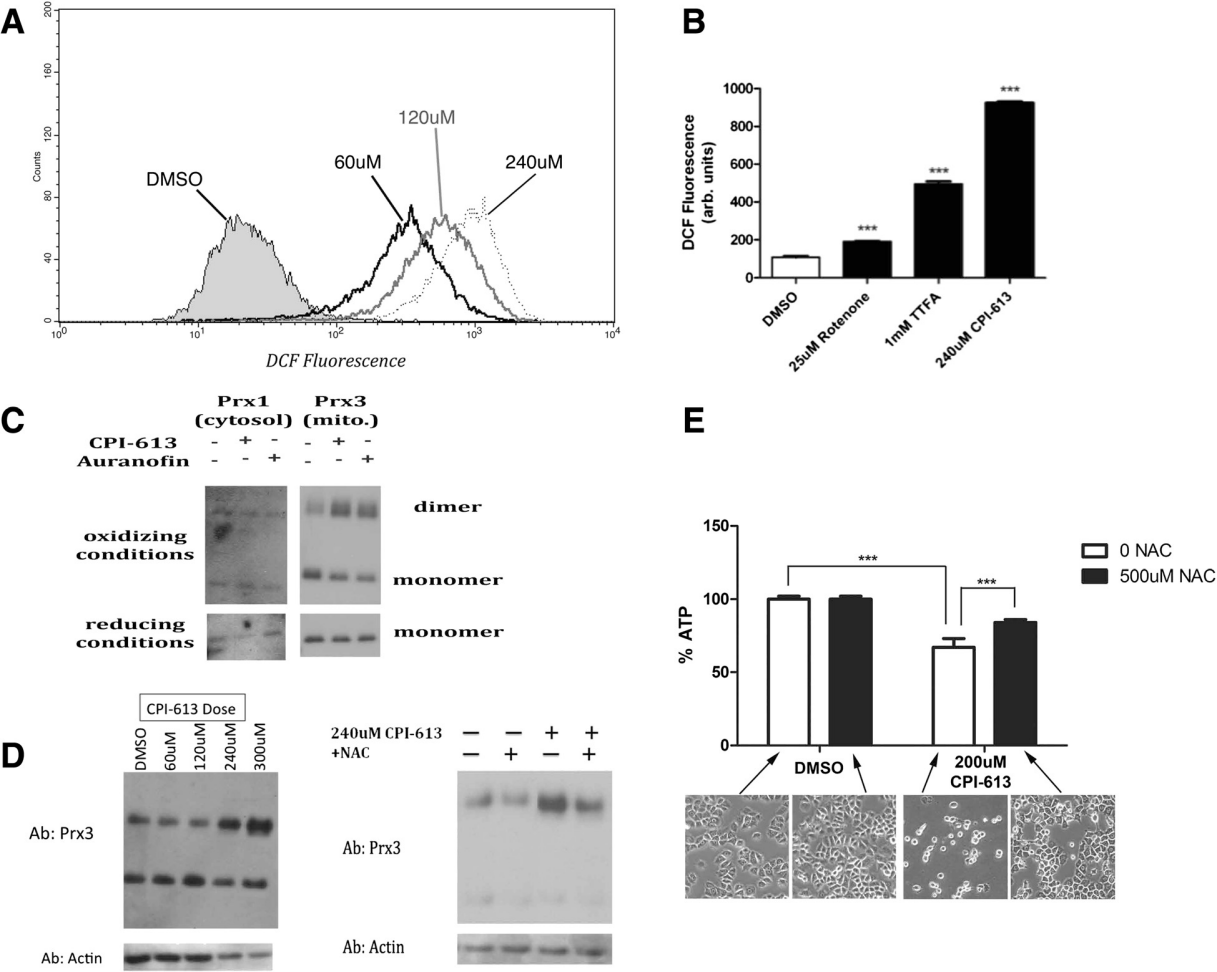
**CPI-613 induces a strong mitochondrial burst of reactive oxygen species implicated in cell death. (A, B)** Intracellular ROS levels were assayed using the hydrogen peroxide-sensitive dye DCF followed by FACS quantification. CPI-613 treatment causes a dose-dependent increase in whole cell DCF signal several-fold higher **(A)** than that caused by classical ROS inducing agents such as rotenone and TTFA at their conventional doses **(B)**. Results are representative of three experiments. (***P <0.0005 compared to vehicle control; Student’s t test; n = 3). **(C)** Upper panel: Levels of dimerization (oxidization) of the cytosolic (Prx1) and mitochondrial (Prx3) isoforms of peroxiredoxin induced by CPI-613 or the mitochondrial inhibitor of ROS detoxification, auranofin, as a control were assayed by western blot (under oxidizing gel conditions). Lower panel: parallel samples were treated with 100 mM DTT and run under reducing gel conditions (converting all peroxiredoxin to the reduced monomer form and serving as a loading control). **(D)** CPI-613-induced Prx3 dimerization is dose dependent (left) and inhibited by co-treatment of cells with the antioxidant NAC (right). **(E)** NAC protects from cell death induced by CPI-613 as assayed by long-term ATP levels (16 hours) and cell morphology. Error bars represent SEM. DCF, 2',7'-dichlorodihydrofluorescein diacetate; DMSO, dimethyl sulfoxide; DTT, dithiothreitol; NAC, N-acetylcysteine; TTFA, thenoyltrifluoroacetone.

To further explore the metabolic and cell death effects produced by CPI-613 treatment, we initially investigated production of ROS, which have emerged as regulatory effectors of both phenomena [31]. Using the ROS-sensitive, cell permeant dye DCF, we observed a robust dose-dependent increase in intracellular ROS levels in H460 human lung carcinoma cells treated with CPI-613 (Figure 2A). Moreover, the amount of ROS produced in response to CPI-613 was several-fold higher than that produced by traditional mitochondrial ROS inducers such as rotenone and thenoyltrifluoroacetone delivered under conventional conditions (Figure 2B).

Mitochondria are a major source of intracellular ROS [31]. To test whether mitochondria were the source of CPI-613-generated ROS, we compared the redox status of the mitochondrial and cytosolic cellular compartments by monitoring oxidation of compartment-specific isoforms of the peroxiredoxin antioxidant proteins [32] in response to acute drug exposure. Initial treatment of H460 cells with CPI-613 caused an increase in the oxidized dimer of the mitochondrial Prx3 isoform with no corresponding increase for the cytosolic Prx1 isoform (Figure 2C). The increase in Prx3 dimerization was dose dependent (Figure 2D) and inhibited by the antioxidant NAC (Figure 2D). Furthermore, NAC also significantly protected cells from drug-induced death as assayed by whole cell ATP levels and cell morphology after 16 hours of drug treatment (Figure 2E). Taken together, these data indicate that mitochondria are the source of CPI-613-induced ROS and implicate this ROS as contributing to CPI-613-induced cell death.

### Electron transport chain complexes I and III are not the source of CPI-613-induced mitochondrial reactive oxygen species

**Figure 3:**
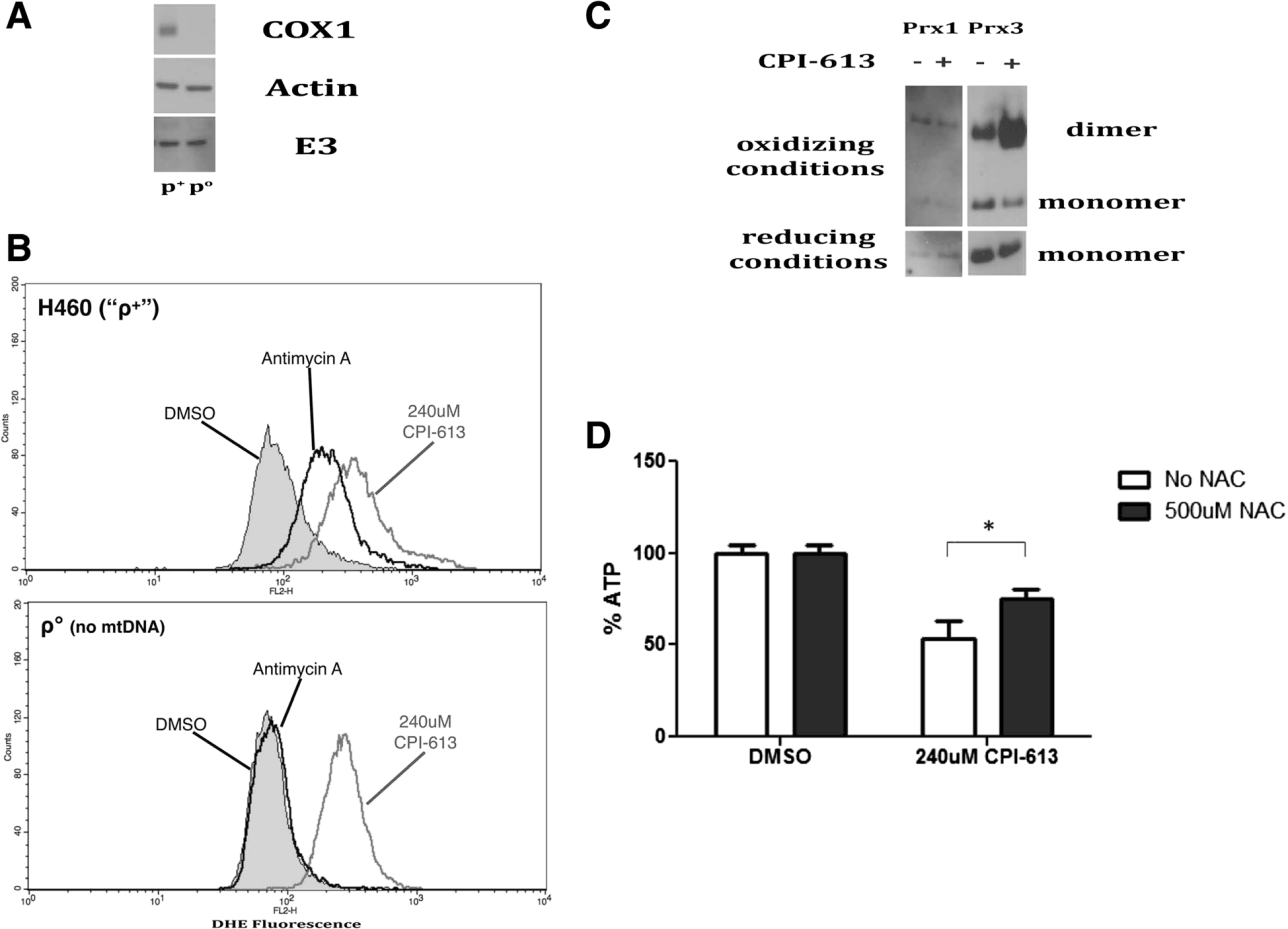
**CPI-613-induced mitochondrial reactive oxygen species originate from a non-electron transport chain source. (A)** ρ° cells lacking mtDNA-encoded components of the ETC were generated from H460 (ρ^+^) as described previously [26] and validated by western blot, demonstrating the absence of the mtDNA-encoded protein cytochrome c oxidase subunit 1 (COX1) but containing nuclear-encoded mitochondrial protein dihydrolipoamide dehydrogenase (E3). The nuclear-encoded cytosolic actin protein served as a loading control. **(B)** ROS levels were quantified following treatment with 240 μM CPI-613 or 4 μM antimycin-A using the superoxide-detecting dye DHE followed by FACS analysis. CPI-613 induced comparable amounts of ROS in ρ° and ρ^+^ cells whereas the complex III ROS inducer antimycin-A failed to increase DHE fluorescence in ρ° cells. Results are representative of three experiments. **(C)** ρ° H460 cells were assayed for oxidation of the mitochondrial Prx3 protein comparably to ρ^+^ in Figure 2C. These ρ° cells produced high levels of mitochondrial ROS by this assay despite lacking key ETC components. **(D)** The antioxidant NAC protects from CPI-613-induced cell death in ρ° cells as assayed by whole cell ATP levels after 20 hours of drug treatment. Error bars represent SEM. DHE, dihydroethidium; DMSO, dimethyl sulfoxide; mtDNA, mitochondrial DNA; NAC, N-acetylcysteine.

Mitochondrial ROS are traditionally associated with perturbation of electron flow through complexes I and III of the electron transport chain (ETC). To investigate the involvement of the ETC in CPI-613-induced ROS and metabolic effects, we generated ρ°-H460 cells that lacked several essential mitochondrial-encoded components of the ETC, and thus generate neither mitochondrial ATP nor ETC-associated ROS from complexes I or III [33,34]. We validated the ρ°-H460 cells by examining the levels of mitochondrial DNA (mtDNA)-encoded proteins. Figure 3A shows no detectable levels of the mtDNA-encoded protein cytochrome c oxidase subunit I in these ρ° cells, whereas the nuclear-encoded proteins actin and dihydrolipoyl dehydrogenase (E3) were at comparable levels to parental (ρ^+^) H460 cells, as expected. Further, these cells could no longer produce ROS in response to the ETC complex III inhibitor antimycin-A as assessed by DHE oxidation (Figure 3B), further confirming their ρ° status. Finally, ρ° status also substantially attenuated ROS production by the ETC complex I inhibitor rotenone without a corresponding effect on CPI-613-induced ROS by this same assay (Additional file 2).

By contrast, ρ°–H460 cells continued to show a large increase in ROS levels when treated with CPI-613. The magnitude of this increase is similar to that seen in ρ^+^-H460 cells (Figure 3B). Additionally, ρ° cells treated with CPI-613 displayed increased oxidized mitochondrial Prx3 levels (Figure 3C) and had similar sensitivity to drug-induced cell death as the parental (ρ^+^) H460 cells (results not shown). These results indicate little or no involvement of the ETC complexes I or III in CPI-613-induced ROS production and cell death. Finally, NAC also protected ρ°-H460 cells from drug-induced cell death (compare Figures 2E and 3D), indicating that ROS-dependent effects originating from some mitochondrial source other than complexes I or III contribute to CPI-613-induced cell death.

### E3 is a major source of the CPI-613 stimulated reactive oxygen species signal

**Figure 4:**
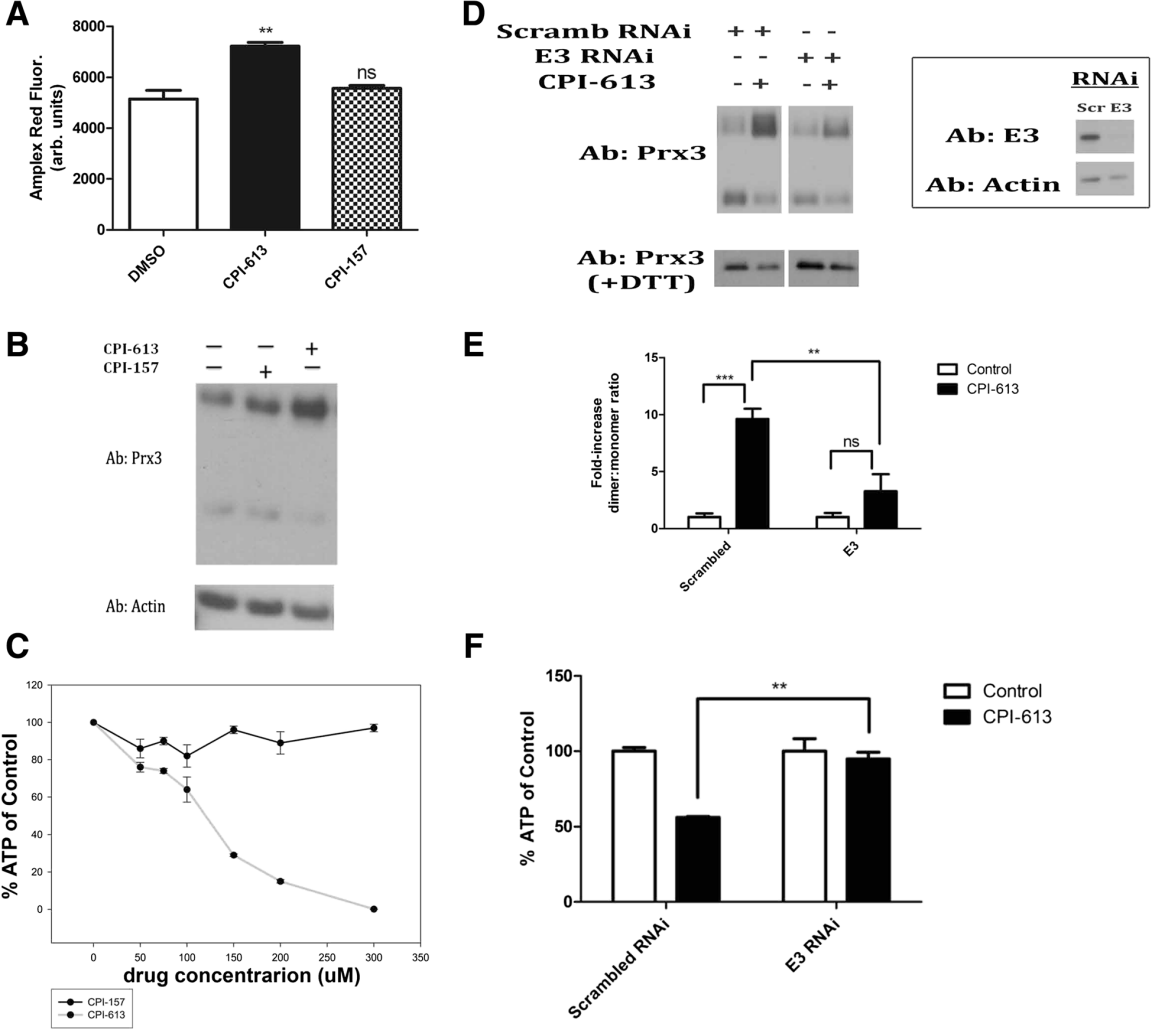
**Analysis of generation and effects of hydrogen peroxide by α-ketoglutarate dehydrogenase *in vivo *and *in vitro*. (A)***In vitro* generation of H_2_O_2_ by KGDH was quantified using Amplex Red oxidation. Co-incubation of KGDH with CPI-613 increased H_2_O_2_ generation by KGDH *in vitro*. CPI-157, a lipoate analog lacking *in vivo* anti-cancer activity, was used as a negative control (see panels **B** and **C**). CPI-157 failed to increase *in vitro* KGDH ROS production. Data are representative of three independent experiments. (**P <0.005 compared to control, ns = not significant compared to control; Student’s t-test; n = 3). **(B)** CPI-157 stimulates mitochondrial H_2_O_2_ generation poorly in treated cells as assessed by Prx3 oxidation. **(C)** CPI-157 is an inactive lipoate analog as assessed by its limited capacity to kill tumor cells. **(D)** Following siRNA-mediated knockdown of the E3 (dihydrolipoamide dehydrogenase) subunit, H460 cells were exposed to 240 μM CPI-613 for 3 hours and Prx3 oxidation was assayed (left). Assessment of E3 protein levels in siRNA treated cells demonstrates efficient knockdown (right). **(E)** Quantification of dimer:monomer ratios in panel **D** using NIH Image-J software **P <0.005 (Student’s t test; n = 3); ***P <0.0005 (Student’s t test; n = 3); ns = not significant. **(F)** H460 cells treated for 16 hours with 240 μM CPI-613 following siRNA knockdown of E3 were assayed for ATP content using Cell-TiterGlo Kit (Promega). ATP loss under these conditions is diagnostic of cell death [18]. Data are expressed as percent of DMSO control. **P <0.005 (Student’s t test; n = 3). All results representative of at least three experiments. Error bars represent SEM. DMSO, dimethyl sulfoxide; DTT, dithiothreitol.

The dihydrolipoyl dehydrogenase (E3 subunit) of KGDH has been identified as a major, non-ETC generator of mitochondrial ROS [22,23]. We used the hydrogen peroxide-detecting dye Amplex Red to examine the effects of CPI-613 on hydrogen peroxide production by purified porcine heart KGDH. CPI-613 treatment resulted in an increase in hydrogen peroxide production, suggesting that KGDH might be a source of the *in vivo* CPI-613 mitochondrial ROS signal (Figure 4A). Note that changes in fluorescence shown in Figure 4A are not the result of direct drug effects on the Amplex Red assay system, as demonstrated by controls in which KGDH is absent (Additional file 3). Furthermore, the related lipoate analog, CPI-157 (Figure 1C), which does not generate significant ROS in cells (Figure 4B) and kills tumor cells poorly (Figure 4C), failed to increase *in vitro* KGDH ROS production. Collectively, these observations corroborate KGDH E3 as a possible *in vivo* source of CPI-613-induced ROS.

To test this hypothesis directly, we used siRNA to knockdown E3 protein (Figure 4D). Following knockdown of E3 levels to <10% of endogenous levels, we observed a significant decrease in mitochondrial ROS (as assayed by Prx3 dimerization) following drug treatment (Figure 4D, quantified in 4E). This observation strongly supports the hypothesis that the E3 of the mitochondrial dehydrogenase complexes is an important source of CPI-613-induced ROS. Finally, E3 knockdown significantly protected from CPI-613-induced cell death (Figure 4F) after 16 hours of drug treatment.

The partial protection from cell death in this experiment coupled with our earlier observation of a similar, partial protection when the PDKs are knocked down [18] collectively indicate that CPI-613 effects on both PDH and KGDH contribute to drug-induced cell death in H460 cells. More specifically, each of these protection effects is highly reproducible and statistically significant; however, longer treatment times or higher drug doses overcome protection, as expected if targeting of KGHD and PDH can each make a partial contribution to CPI-613-induced cell death.

### Tumor cell α-ketoglutarate dehydrogenase is inhibited in a redox-dependent manner by CPI-613 treatment

ROS have been shown to regulate a number of cellular metabolic enzymes [3,5,8]. Moreover, several studies indicate that KGDH may be redox regulated (reviewed in [35,36]). These observations suggest the hypothesis that KGDH may not only be a source of the CPI-613 induced ROS signal, but also a target of that signal.

**Figure 5:**
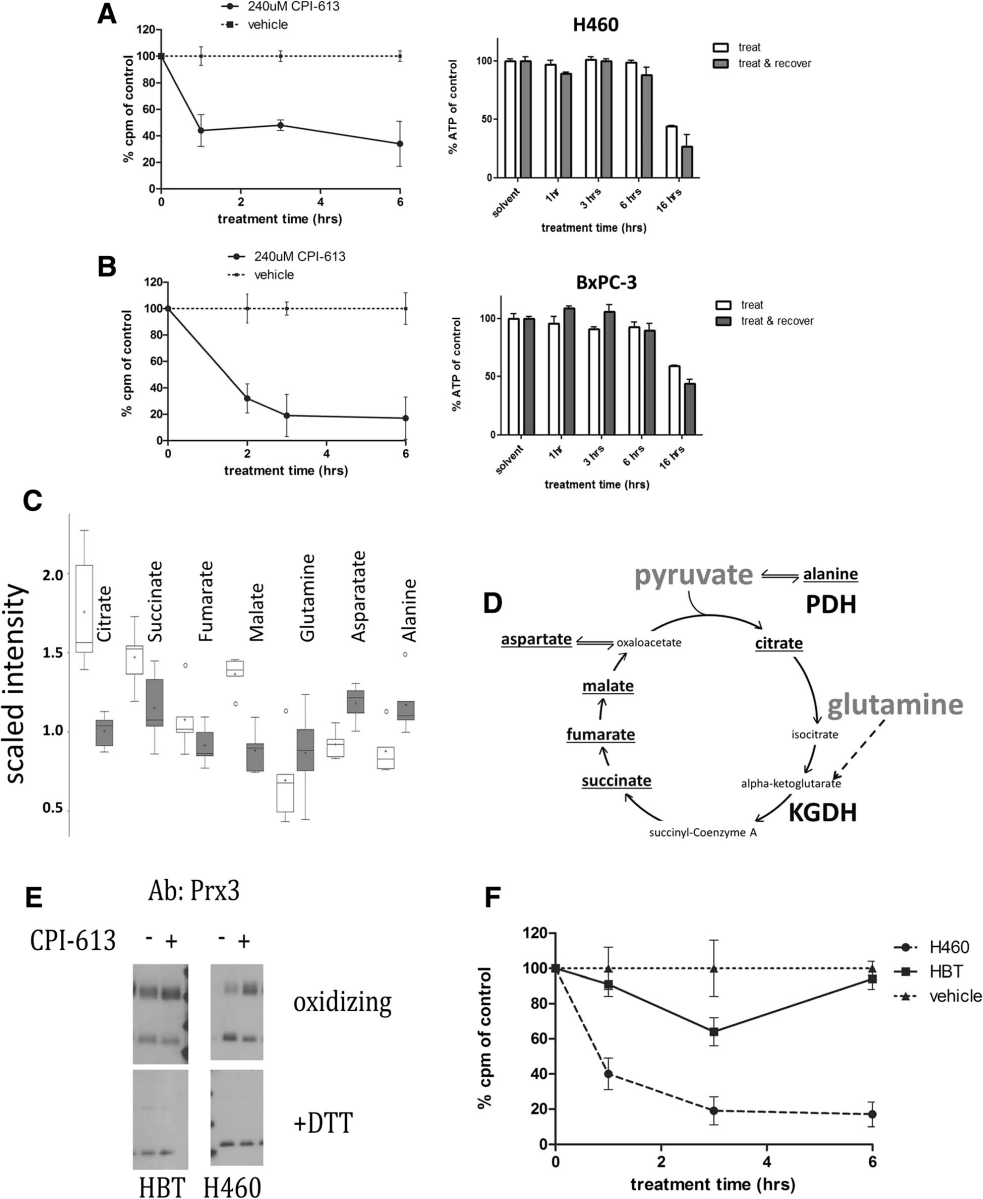
**CPI-613 inhibits α-ketoglutarate dehydrogenase activity selectively in tumor cells. (A, B)** Flux through KGDH was assayed in H460 **(A)** and Bx-PC3 **(B)** tumor cells using pulse delivery of 1-^14^C-labeled glutamate, whose labeled carbon is released as CO_2_ by KGDH. Each flux panel is coupled on the right with a parallel experiment demonstrating that commitment to and execution of cell death (measured by ATP levels, before or after 3 hours of recovery from drug treatment) occurs well after times used for flux analysis. **(C)** Steady-state metabolite analysis of BxPC-3 tumor cells after 2-hour treatment with 240 μM CPI-613 (shaded boxes) compared to mock-treated samples (open boxes) shows decreases inTCA cycle intermediates citrate, succinate, fumarate and malate and increases in anaplerotic inputs alanine, aspartate and glutamine. **(D)** Diagram of the TCA cycle, its two main carbon entry points, and anaplerotic transaminations supporting alanine and aspartate catabolism. **(E, F)** Normal HBT cells show no increase in Prx3 oxidation **(E)** and only a slight, transient inhibition of KGDH flux **(F)** under conditions producing robust effects in lung cancer cells. Vehicle control in panel **F** refers to the tumor cells; the HBT vehicle control behaved similarly. All results are representative of at least three experiments or (panel **C**) data point collections. Error bars represent SEM except for panel **C** in which they represent 95% confidence limits. Box plots (panel **C**) are used to convey the spread of the data with the middle 50 % of the data represented by boxes and whiskers reporting the range of the data. The solid bar across the box represents the median value of those measured while the + is the mean. Any outliers are shown as dots outside the whiskers of the plot. DTT, dithiothreitol; HBT cells, primary human bronchial/tracheal epithelial cells; KGDH, α-ketoglutarate dehydrogenase.

To investigate the effects of CPI-613 on KGDH activity, we examined carbon flux through KGDH by monitoring CO_2_ release from cells pulse-labeled with 1-^14^C-glutamate. Glutamate is converted to α-ketoglutarate in the mitochondrion and enters the TCA cycle through oxidative decarboxylation by KGDH, resulting in the release of the 1-carbon as CO_2_. Treatment of cells with CPI-613 results in a large reduction in radiolabeled CO_2_ release after a 1-^14^C-glutamate pulse in both H460 lung carcinoma (Figure 5A) and BxPC-3 (Figure 5B) pancreatic carcinoma cells, indicating that CPI-613 inhibits KGDH activity. Note that drug-induced cell death makes a negligible contribution to the reduction in KGDH activity at these short treatment times (Figure 5A,B).

To further corroborate inhibition of KGDH activity by CPI-613, we carried out a steady-state metabolomics analysis in BxPC-3 cells in collaboration with Metabolon, Inc. Treatment of BxPC-3 cells with CPI-613 resulted in reduction of the levels of succinate, fumarate and malate, TCA cycle intermediates downstream of KGDH, as expected if KGDH is inhibited by drug treatment (Figure 5C,D). Though levels of substrates α-ketoglutarate and pyruvate were too low to measure in these experiments, glutamine (a proxy for KGDH substrate) showed scaled intensity levels 25% higher in treated cells, corroborating KGDH inhibition. As a more general control for the decrease in TCA cycle intermediates, we observed the expected elevation in diverse metabolites whose catabolism depends on the TCA cycle (for example, 33% elevation for alanine and 28% for aspartate). Note that citrate levels were also reduced, consistent with the known inhibition of PDH activity by CPI-613 [18]. This reduction in citrate levels is unlikely to be the cause of reduced succinate, fumarate and malate levels in view of the anaplerotic input from glutamine through KGDH.

We previously demonstrated significant tumor cell selectivity of CPI-613 effects, showing that drug inhibition of PDH by over-stimulation of regulatory phosphorylation was highly selective for tumor cells, correlating with significant selectivity for cell-death induction in tumor cells (see Figure 4B in [18]). Here, we add important new pieces of evidence for CPI-613 tumor cell selectivity. Specifically, robust Prx3 oxidation and KGDH flux inhibition by CPI-613 were not seen in the normal, primary human bronchial epithelial cell line HBT (a non-malignant control for H460 lung tumor cells) in response to CPI-613 (Figure 5E,F).

To assess the possibility that these results reflect a broad loss of mitochondrial function, rather than specific inhibition of KGDH, we investigated acute effects of CPI-613 on energy production by fatty acid oxidation. This process yields substantial mitochondrial ATP independently of the TCA cycle, whose function is compromised by KGDH and PDH inactivation. Specifically, initial mitochondrial beta oxidation of fatty acids delivers reducing equivalents directly to the electron transport system. The resulting acetate units (acetyl-CoA) are excreted as citrate allowing cyclic regeneration of free CoA through the citrate synthase reaction. The citrate synthase reaction is provided with its other necessary substrate, oxaloacetate, through transamination of media-provided aspartate.

**Figure 6:**
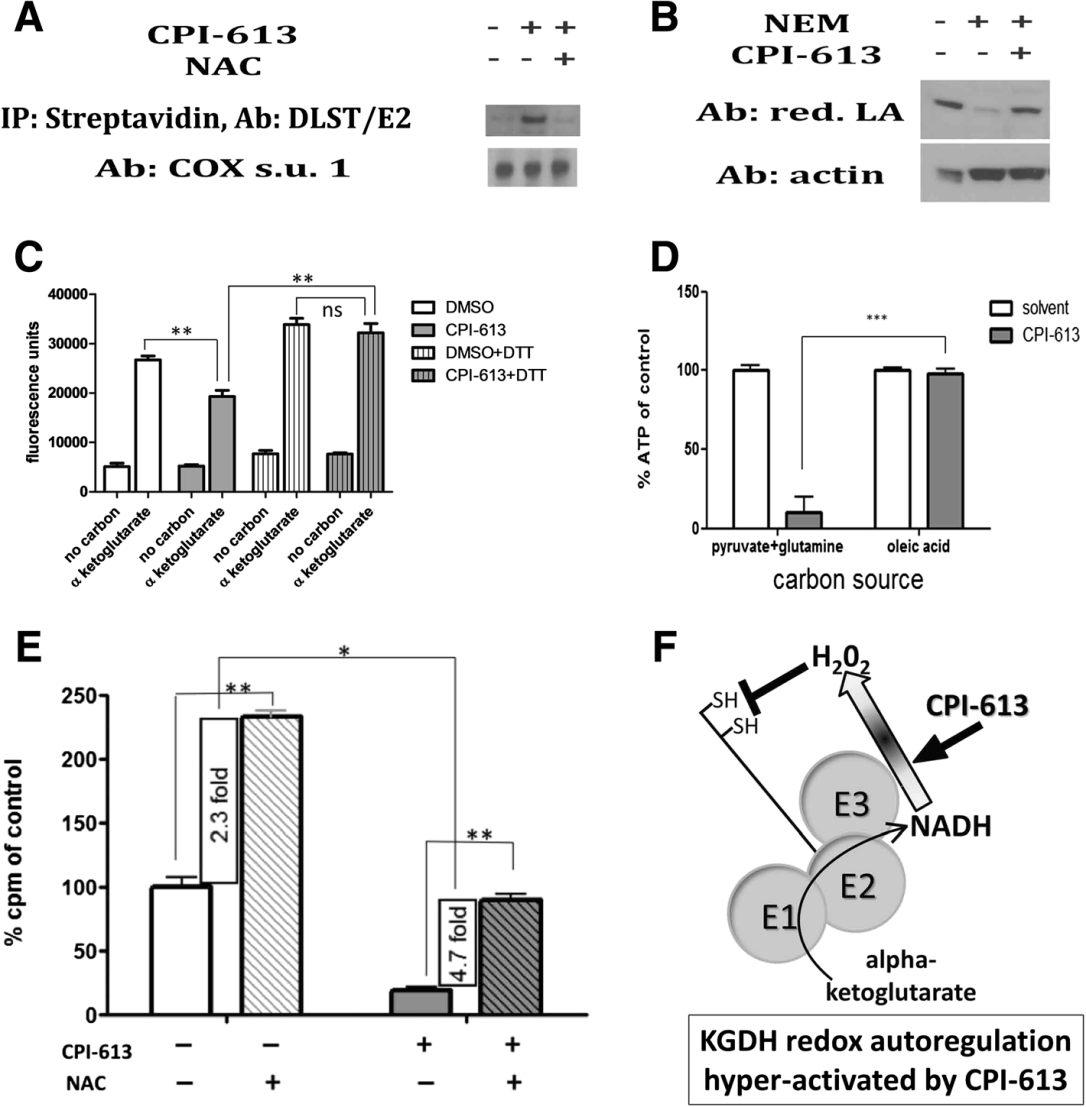
**CPI-613 induces reactive oxygen species-mediated glutathionylation and inhibition of tumor cell α-ketoglutarate dehydrogenase. (A)** KGDH E2 enriched in CPI-613-treated cells following capture of glutathionylated proteins using biotin-switch (text). KGDH glutathionylation suppressed by co-treatment with 250 μM NAC. **(B)** Cells were treated with 240 μM CPI-613 then exposed to 100 mM NEM followed by 100 mM DTT, chemically reversing redox modifications. Western blot analysis of native KGDH lipoate indicates increased levels of NEM-protected residues in CPI-613-treated samples. Thus, KGDH lipoate sulfurs are a target of drug-induced redox modification. **(C)** α-ketoglutarate produces robust *in vitro* NAD^+^ reduction and this process is significantly inhibited in CPI-613-treated cells. This inhibition is relieved by 10 mM DTT treatment of the lysates. Thus, KGDH from CPI-613-treated cells is inhibited by redox modification. **(D)** CPI-613 (2 hours at 240 μM) selectively inhibits mitochondrial ATP production driven by PDH and KGDH substrates, pyruvate and glutamine, but not driven by fatty acid oxidation. **(E)** 500 μM NAC increases carbon flux through KGDH, suggesting a role for H_2_O_2_ in KGDH regulation in H460 tumor cells. NAC treatment strongly reverses the inhibition of KGDH activity by CPI-613. The magnitude of this effect (4.7-fold) is greater than the increase in carbon flux in untreated cells (2.3-fold), indicating that NAC acts to reverse CPI-613 inhibition of KGDH activity in addition to its effects on KGDH regulation in absence of drug. **(F)** Proposed model of mechanism of action of CPI-613 on KGDH. CPI-613 may ‘misinform’ existing tumor cell KGDH redox autoregulation (shaded block arrow), increasing this ROS signal generated by the E3 subunit (including from the reverse reaction, NADH oxidation), resulting in E2-subinit redox modification and KGDH inhibition. All results are representative of at least three independent experiments. Error bars represent SEM. DMSO, dimethyl sulfoxide; DTT, dithiothreitol; KGDH, α-ketoglutarate dehydrogenase; NAC, N-acetylcysteine; NEM, N-ethylmaleimide.

To examine the status of these functions, we exploited our previous demonstration that mitochondrial ATP synthesis can be examined directly when cells are provided with exclusively mitochondrial carbon sources (no glucose) [18]. As expected, mitochondrial ATP synthesis in H460 cells sustained by TCA cycle-dependent substrates (pyruvate and glutamine) was rapidly and catastrophically inhibited by CPI-613 treatment (Figure 6D). By contrast, when these cells were provided with a fatty acid (oleic acid) as the sole major carbon source, acute drug treatment consistently produces little or no effect on ATP synthesis (Figure 6D).

This result demonstrates that substantial segments of mitochondrial energy metabolism external to the TCA cycle remain functional (including the beta-oxidation machinery, the electron transport system, and the ATP synthase), consistent with CPI-613 effects being restricted to specific targets, including KGDH.

### CPI-613 induces reactive oxygen species-mediated inactivation and glutathionylation of α-ketoglutarate dehydrogenase

The dihydrolipoamide succinyltransferase (E2) subunit of KGDH contains sulfurs with the characteristics expected of redox-sensitive targets, including those of the enzyme’s lipoates (Figure 1). When exposed to ROS, these sulfurs are vulnerable to oxidative modifications, including those culminating in glutathionylation. Glutathionylation of lipoate sulfhydryls is associated with enzymatic inhibition in response to ROS exposure [24]. To test whether ROS-induced glutathionylation occurred in response to CPI-613 treatment, we used a modification of the biotin-switch assay to enrich for glutathionylated proteins [21]. Following CPI-613 treatment we observed a large increase in glutathionylation levels of the E2 subunit of KGDH (Figure 6A). Notably, this increase in KGDH E2 glutathionylation was prevented by NAC treatment, providing further evidence for the direct involvement of ROS in CPI-613 modification of KGDH activity.

In addition to the sulfurs of lipoate, the E2 subunit contains multiple cysteine residues that could also be susceptible to glutathionylation. To test whether the lipoate residues of KGDH might be targets of drug-induced redox modification, we took advantage of antibodies recognizing native lipoate but not chemically modified lipoate. Glutathionylation and/or other redox modifications protect lipoate sulfurs from chemical derivatization by the alkylating agent NEM. CPI-613 treatment resulted in significantly fewer alkylated lipoate residues following NEM exposure (Figure 6B). This result demonstrates that KGDH lipoate residues acquire reversible, redox-sensitive modifications (consisting of glutathionylation and/or other derivatizations) expected to block KGDH E2 activity as a result of CPI-613 treatment.

To confirm that CPI-613 directly inhibits KGDH, we examined enzyme activity in lysates from treated cells. While the labile status of redox protein modifications is sufficiently high that we do not expect enzymes in lysates to fully recapitulate *in vivo* effects, we nonetheless explored whether we could retain measureable KGDH modification *in vitro*. As expected, CPI-613 treatment of H460 cells produced a significant, reproducible reduction in KGDH activity in the resulting lysates (Figure 6C). Moreover, this drug-induced KGDH inhibition was eliminated by treatment of the lysates with the reducing agent, DTT (Figure 6C). This result confirms that CPI-613 effects include direct inhibition of KGDH activity in a redox-dependent fashion.

The small super-induction of KGDH activity by DTT treatment in this study is reproducible and suggestive. We explore its significance in the more robust *in vivo* environment in the following section.

### Evidence for redox autoregulation of α-ketoglutarate dehydrogenase activity

The results above indicate that KGDH is both a source and a target of CPI-613-induced ROS. This introduces the possibility that KGDH is autoregulated in a redox-dependent fashion (at least in tumor cells) and that CPI-613 perturbs this process. To test this hypothesis, we examined the effect of NAC on carbon flux through KGDH, independent of drug treatment, utilizing the 1-^14^C-glutamate oxidation assay (as above). Figure 6E shows that flux through KGDH was significantly elevated by the presence of NAC in non-drug-treated H460 tumor cells. Furthermore, NAC treatment substantially protected against KGDH flux inhibition induced by CPI-613. Note especially that the magnitude of NAC protection from CPI-613 effects was substantially greater than the NAC effects on flux through KGDH in untreated cells. These results indicate that NAC protects cells from the action of CPI-613 on KGDH activity, in addition to its effects on KGDH regulation in the absence of drug.

Collectively, these results strongly suggest that ROS play a significant role in the regulation of KGDH in tumor cells and that CPI-613 interacts with this regulation in a manner that results in strong additional redox inhibition of KGDH activity (Figure 6F).

## Discussion

We report new insights into the anti-cancer mechanism of action of CPI-613, a member of a novel lipoate analog agent class. Our definition of a new drug target here, together with our earlier work [18], indicate that CPI-613 simultaneously attacks two pivotal tumor mitochondrial metabolic enzymes, each through a distinct proximate mechanism. Moreover, collectively, these two CPI-613 targets, PDH and KGDH, control the majority of the carbon flux through the TCA cycle in most tumor cells. Finally, the KGDH and PDH regulatory targets of these agents appear to behave substantially differently in tumor cells than in normal cells, conferring significant tumor selectivity on CPI-613.

ROS are intimately associated with mitochondrial function and dysfunction. Moreover, redox signaling is extensively altered in tumor cells (reviewed in [36-39]). We observed a large burst of ROS upon CPI-613 treatment of tumor cells localized to mitochondria. The primary source of this mitochondrial ROS burst was not complex I or III of the ETC, as ρ° cells lacking the capacity for ROS generation from these sources displayed a comparable ROS surge. Rather, CPI-613-induced ROS generation was largely or entirely dependent upon the dihydrolipoamide dehydrogenase (E3) component of mitochondrial dehydrogenase complexes, including KGDH.

Consistent with this drug-induced E3 ROS generation playing a role in cell death, E3 RNAi knockdown significantly attenuates mitochondrial ROS production and cell death following CPI-613 treatment. Concomitant with KGDH ROS generation, we observed a marked decrease in KGDH enzymatic activity that was prevented by co-treatment with the antioxidant NAC, indicating ROS-induced inhibition of KGDH. We also show that redox modification of KGDH correlated with inhibition of this enzyme’s activity, both in cultured cells and in cell lysates from treated cells. KGDH was strongly glutathionylated in response to CPI-613 treatment and KGDH lipoates were modified in a redox-sensitive manner. Glutathionylation of these lipoate sulfhydryls has been previously demonstrated in response to direct treatment of respiring mitochondria with hydrogen peroxide and these modifications block KGDH function as expected [21]. The redox modifications of KGDH lipoates we observed may or may not include glutathionylation; however, any redox modification of these lipoates is expected to block KGDH E2 function.

The mechanism of KGDH ROS generation is not completely understood. However, the E3 subunit is known to act in both the physiological (forward, reduction of NAD^+^ to NADH) and reverse (oxidation of NADH to reduce lipoamide to dihydrolipoamide) directions. The work of Ambrus and collaborators indicates that, under conditions where normal substrates such as NAD^+^ are present at unusually low levels, the FAD domain of E3 will, instead, transfer electrons to molecular oxygen. This generates superoxide, which is, in turn, rapidly dismutated to hydrogen peroxide spontaneously and through mitochondrial superoxide dismutase activity [40]. Moreover, reducing potential (reduced lipoamide or NADH) from either the forward or reverse reactions, respectively, can be used by E3 to generate superoxide or hydrogen peroxide [35]. Collectively, these results indicate that both the oxidation and reduction status of KGDH lipoates and the E3 generation of hydrogen peroxide are sensitive reflections of the energy status of the mitochondrial matrix.

In view of these observations, our data strongly suggest that KGDH ROS generation is the basis of an endogenous autoregulatory mechanism in its own right (at least in tumor cells). Given KGDH’s pivotal role in the TCA cycle, it is reasonable to expect diverse mechanisms regulating this enzymatic activity.

Like allosteric regulation, redox regulation can provide real-time information about bio-availability of a substrate or product, allowing direct modulation of enzymatic activity in response. As mentioned above, given the central role of lipoate acylation and oxidation and reduction in the KGDH catalytic cycle, it is likely that ratios of these lipoate intermediates are a rich source of useful regulatory information, as they are known to be in the case of PDH (reviewed in [18,19]).

Our data strongly suggest the hypothesis that CPI-613 (a lipoate analog) ‘misinforms’ a lipoate-state-responsive redox regulatory process controlling KGDH in such a fashion as to drive increased production of ROS catalyzed by the E3 subunit. The consequences of this misinformation include the ROS-induced glutathionylation of KGDH E2 and down-modulation of KGDH activity (Figure 6F). In this autoregulation hypothesis, the absence of CPI-613 (that is, under physiological conditions) would result in steady-state KGDH ROS production that would feedback to reversibly inhibit KGDH activity (through redox modification of KGDH lipoates and possibly other sulfhydryls) in response to excess reducing potential production, down-regulating flux to a set point defined by the kinetic properties of the circuit, thereby acting as a continuous governor of carbon flow through KGDH.

In our working hypothesis, this feedback modulation of ROS production is stimulated by the presence of the non-redox-active CPI-613 analog. For example, ROS production by E3 can reasonably be expected to be allosterically responsive to the acylation and/or redox status of neighboring E2 lipoates (consistent with drug effects on purified KGDH; Figure 4A), constituting a target for the action of lipoate analogs. Such drug modulation of redox autoregulation is a plausible detailed mechanism for the contribution of KGDH inhibition to the acute tumor mitochondrial metabolic collapse observed within the first hour of treatment, ultimately resulting in commitment to cell death [18]. If CPI-613 is removed by washout within 3 hours, cells recover and survive [18], despite the large amounts of mitochondrial ROS produced during initial exposure to the drug. This is the expected behavior if an evolved function of this ROS production includes metabolic regulation rather than immediate induction of cell death.

The tumor specificity of CPI-613 redox effects suggests that components of the KGDH redox regulatory process itself (or its immediate context) are altered in some way(s) in tumor cells. Together with the recent discovery of direct redox effects on cytosolic glycolytic flux via cysteine oxidation in the tumor-specific pyruvate kinase M2 isoform [8], these observations corroborate the emerging view that the altered redox regulatory state of cancer metabolism might comprise a useful set of targets for chemotherapy. Owing to its simultaneous tumor-specific targeting of a second gate-keeping enzyme (PDH) in a mechanistically distinct fashion, CPI-613 targeting of KGDH may hold particular promise in this cancer redox-matter/energy metabolism niche. By targeting two tumor-specific activities, CPI-613 apparently behaves as a treatment ‘cocktail of one.’ It will be of considerable interest to explore the molecular determinants of these apparent KGDH regulatory alterations in more detail in the future.

Finally, the mitochondrial redox signal induced by CPI-613 is quite powerful, as evidenced by the extent of Prx3 oxidation. Thus, it will be of great interest to explore possible ROS targets beyond KGDH. Of particular interest will be the other lipoate-using, E3-containing mitochondrial complexes (PDH; branched chain alpha-keto acid dehydrogenase; and the glycine cleavage system). For example, while our earlier results demonstrate that tumor-specific kinase (PDK) regulation of PDH is a primary proximate target for CPI-613 action on this enzyme [18], it remains possible that redox regulation might play a secondary role in drug effects on PDH.

## Conclusions

CPI-613 induces redox-mediated inactivation of the pivotal TCA cycle enzyme, KGDH, selectively in tumor cells. This inhibition of an enzyme vital to energy flow is associated with the catastrophic inhibition of tumor mitochondrial metabolism by this drug, followed by tumor cell death. The CPI-613-induced inactivation is associated with redox modification of the endogenous KGDH lipoates of the E2 subunit, apparently in response to KGDH E3-generated ROS. This novel mechanism of action indicates a previously unsuspected reprograming of KGDH regulation in tumor cells, including redox autoregulation. These insights add strong impetus to the newly emerging insight that targeting the altered redox regulation of matter/energy metabolism in tumor cells may be an especially attractive source of chemotherapeutic targets. Moreover, we have previously reported that CPI-613 simultaneously attacks the other major entry point for carbon into the typical tumor mitochondrial TCA cycle, the lipoate-using enzyme, PDH, using a distinct, non-redox mechanism. Collectively these results indicate that CPI-613 simultaneously attacks at least two cancer metabolic targets, independently, a unique feature that indicates the possibility of strong clinical potential and a new opportunity to target cancer by attacking metabolic regulation [41].

## Abbreviations

CoA: coenzyme A; DCF: 2',7'-dichlorodihydrofluorescein diacetate; DHE: dihydroethidium; DTT: dithiothreitol; EDTA: ethylenediaminetetraacetic acid; EGTA: ethyleneglycoltetraacetic acid; ETC: electron transport chain; FACS: fluorescence-activated cell sorting; HBT: human bronchial/tracheal epithelial cells; HEPES: 4-(2-hydroxyethyl)-1-piperazineethanesulfonic acid; KGDH: alpha-ketoglutarate dehydrogenase; LDS: lithium dodecyl sulfate; mtDNA: mitochondrial DNA; NAC: N-acetylcysteine; NEM: N-ethylmaleimide; PDH: pyruvate dehydrogenase; PDK: pyruvate dehydrogenase kinase; ROS: Reactive oxygen species; RPMI: Roswell Park Memorial Institute; siRNA: small interfering RNA; TCA: tricarboxylic acid.

## Competing interests

ADK and BRK have no competing interests. SDS, AS and SG are or were employed by Cornerstone Pharmaceuticals, Inc. ZZ and PMB have a financial interest in Cornerstone Pharmaceuticals, Inc.

## Authors’ contributions

SDS contributed to design and interpretation of all experiments, drafting of the manuscript and execution of assessment of ROS production, redox modification of KGDH, E3 RNAi effects on ROS production, and *in vitro* inhibition of KGDH function. AS contributed to the design, execution and interpretation of experiments assessing CPI-613 dose and ROS production, NAC effects on ROS production and cell death, and the characterization of CPI-157. SG contributed to the design and execution of E3 RNAi effects on drug response. ADK and BRK contributed to the design of and executed the steady state metabolomics. ZZ contributed to design and interpretation of all experiments, drafting of the manuscript and execution of flux metabolomics. PMB contributed to design and interpretation of all experiments, and the drafting of the manuscript. All authors assisted with the completion of the manuscript. All authors read and approved the final manuscript.

## Supplementary Material

Additional file 1 Supplemental Methods. Click here for file

Additional file 2: Figure S1 In contrast to CPI-613, rotenone-induced ROS is substantially attenuated in ρ° cells. ROS production was measure by FACS analysis of DHE fluorescence as in Figure 3 in the main text. Note that ρ° cells display substantially reduced fluorescence induced by rotenone (50 μM, 3 hours), while the CPI-613-induced fluorescence is not attenuated in ρ° cells. Click here for file

Additional file 3: Figure S2 CPI-613 and CPI-157 have no effect on the Amplex Red assay system in the absence of KGDH. Shown are results of the Amplex Red fluorescence assay system (Figure 4A) in the absence of the KGDH enzyme. Note that neither of the drugs used in these studies produce significant elevation in the low fluorescence levels produced in the absence of enzyme. Click here for file
