# Supplementary material for: A strategically designed small molecule attacks alpha-ketoglutarate dehydrogenase in tumor cells through a redox process
Source: Cancer Metab. 2014 Mar 10;2:4. doi: 10.1186/2049-3002-2-4 (PMC4108059; doi:10.1186/2049-3002-2-4)
Supplement: Additional file 1 — Supplemental Methods. [file 2049-3002-2-4-S1.docx]

**A strategically designed small molecule attacks alpha-ketoglutarate dehydrogenase in tumor cells through a redox process**

Shawn D. Stuart^1^, Alexandra Schauble^3^, Sunita Gupta^3^,

Adam D. Kennedy^4^, Brian R. Keppler^4^,

Paul M. Bingham^2,3^* and Zuzana Zachar^2,3^

**Supplemental Methods**

**Steady state metabolite levels determination – cell preparation**. Cell preparation was as follows. Briefly, 3.5x10^6^ BxPC-3 pancreatic cancer cells were plated in 10cm plates in RMPI medium supplemented with 10% FBS and 100U/ml penicillin and 100ug/ml streptomycin. Cells were allowed to grow for two days reaching 90-95% confluence. At this time, the medium was replaced with fresh medium containing solvent (0.075%dimethylformamide) or 240μM CPI-613 in solvent and treated for 120 minutes. At the end of treatment, plates were placed on ice, medium was removed and cells were washed with 3mls of ice-cold non-supplemented RMPI medium. 3mls of fresh ice-cold non-supplemented medium was added to the plates, cells were scraped and transferred into 15 ml semiconical tubes and centrifuged at 800xg for 3 minutes. Supernatant was removed, samples were spun for an additional 30 seconds and all residual medium was removed. Cell pellets were flash frozen in liquid Nitrogen and stored at -80°C until shipment to Metabolon for biochemical profiling.

**Steady state metabolite levels determination - global untargeted biochemical profiling**

The LC/MS portion of the platform was based on a Waters ACQUITY UPLC and a Thermo-Finnigan LTQ mass spectrometer, which consisted of an electrospray ionization (ESI) source and linear ion-trap (LIT) mass analyzer.  The sample extract was split into two aliquots, dried, then reconstituted in acidic or basic LC-compatible solvents, each of which contained 11 injection standards at fixed concentrations. One aliquot was analyzed using acidic positive ion optimized conditions and the other using basic negative ion optimized conditions in two independent injections using separate dedicated columns. Extracts reconstituted in acidic conditions were gradient eluted using water and methanol both containing 0.1% Formic acid, while the basic extracts, which also used water/methanol, contained 6.5mM Ammonium Bicarbonate. The MS analysis alternated between MS and data-dependent MS [28] scans using dynamic exclusion. We performed chromatographic separation, followed by full-scan mass spectroscopy, to record and quantify all detectable ions presented in the samples. We identified metabolites with known chemical structure by matching the ions’ chromatographic retention index and mass spectral fragmentation signatures with reference library entries created from authentic standard metabolites under the identical analytical procedure as the experimental samples.

An aliquot of each experimental plasma sample was taken then pooled for the creation of “Client Matrix” (CMTRX) samples. These CMTRX samples were injected throughout the platform run and served as technical replicates, allowing variability in the quantitation of all consistently detected endogenous biochemicals to be determined and overall process variability and platform performance to be monitored.

**Steady state metabolite levels determination - statistical analysis.** Data normalization was performed to correct variation resulting from instrument inter-day tuning differences. Each compound was corrected in run-day blocks by registering the medians to equal one (1.00) and normalizing each data point proportionately. Missing values (if any) were assumed to be below the level of detection for that biochemical with the instrumentation used and were imputed with the observed minimum for that particular biochemical. After normalization and imputation, the data were log-transformed. We then performed *t* tests to compare the treatment conditions as well as the time points across treatments. Multiple comparisons were accounted for with the false discovery (FDR) rate method, and each FDR was estimated by q-values.

**H460 and Bx-PC-3 cells do not commit to drug-induced cell death until after the treatment period used for flux analyses.** Commitment to cell death was assayed in H460 and BxPC-3 cells using Promega CelltiterGlo assay to determine cell viability. Treatment conditions were identical to those used to measure KGDH carbon flux (Figure 5A and B, main text). Cells were grown and treated in RPMI medium (including 11.11mM glucose) with 240uM CPI-613 for times indicated (open columns) and assayed for viability. In parallel samples, at the end of the treatment interval, medium was replaced with fresh medium without drug, cells were allowed to recover for 3 hours, and cell viability was assessed (shaded columns). Under these recovery conditions, cells scoring as viable survive indefinitely, including continued growth after splitting and replating [18]. Note that up to 6 hours of treatment, cell viability remains at above 88% (H460) and 90% (BxPC-3) while KGDH activity has fallen by 60%-80% (Figure 5A, B, and F). Thus, the large majority of the drug-induced reduction in KGDH activity is not the result of cell death. Also included as a positive control is a 16 hr treatment at which time more than 75% (H460) or 65% (BxPC-3) cells have undergone cell death commitment. Error bars represent SEM.
