# Supplementary figures and images for: A strategically designed small molecule attacks alpha-ketoglutarate dehydrogenase in tumor cells through a redox process
Source: Cancer Metab. 2014 Mar 10;2:4. doi: 10.1186/2049-3002-2-4 (PMC4108059; doi:10.1186/2049-3002-2-4)

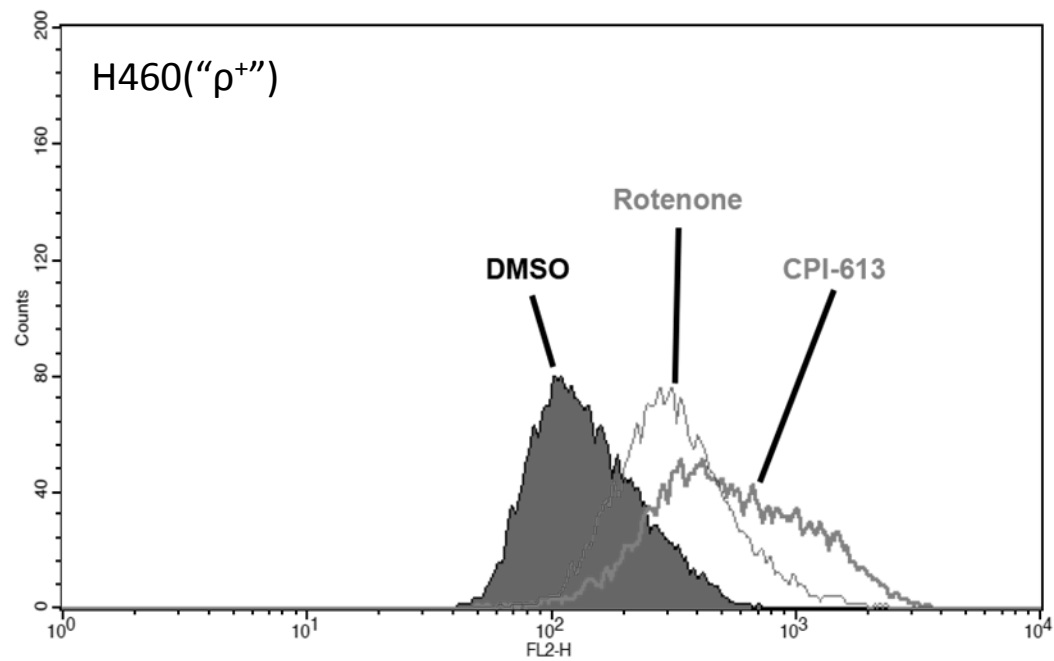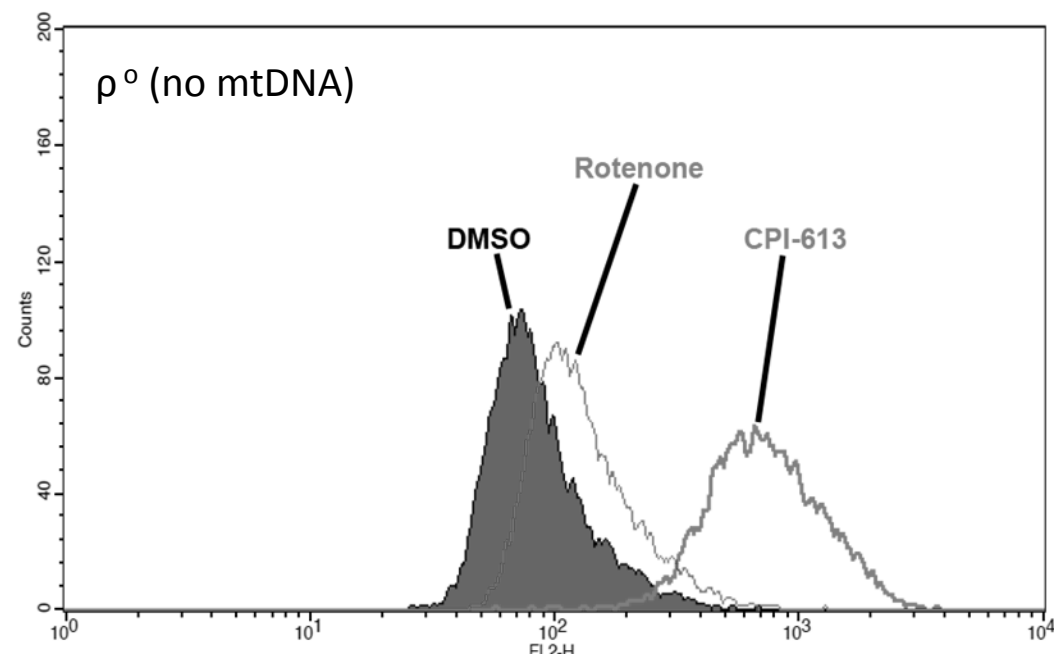

Supplement: Additional file 2: Figure S1 — In contrast to CPI-613, rotenone-induced ROS is substantially attenuated in ρ° cells. ROS production was measure by FACS analysis of DHE fluorescence as in Figure 3 in the main text. Note that ρ° cells display substantially reduced fluorescence induced by rotenone (50 μM, 3 hours), while the CPI-613-induced fluorescence is not attenuated in ρ° cells. [file 2049-3002-2-4-S2.pdf]

# Chemical Effects on Amplex Red Reaction

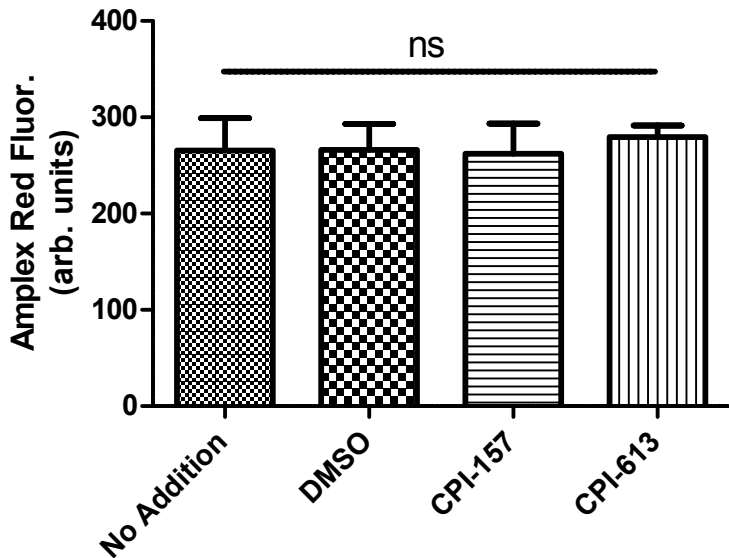

Supplement: Additional file 3: Figure S2 — CPI-613 and CPI-157 have no effect on the Amplex Red assay system in the absence of KGDH. Shown are results of the Amplex Red fluorescence assay system (Figure 4A) in the absence of the KGDH enzyme. Note that neither of the drugs used in these studies produce significant elevation in the low fluorescence levels produced in the absence of enzyme. [file 2049-3002-2-4-S3.pdf]
